# From Food Waste to Food Citizenship via Food Ethics—Cross-Country Analysis †

**DOI:** 10.3390/foods15213822

**Published:** 2026-10-26

**Authors:** Ioana Mihaela Balan, Teodor Ioan Trasca, Nicoleta Mateoc-Sirb

**Affiliations:** 1 University of Life Sciences “King Mihai I”, 30645 Timisoara, Romania; ioanabalan@usvt.ro; 2 University of Agronomic Sciences and Veterinary Medicine of Bucharest, 011464 Bucharest, Romania; 3 Branch of Timisoara, Romanian Academy, 300223 Timisoara, Romania

**Keywords:** food waste, food loss, food ethics, food citizenship, food security, food sustainability, overconsumption, sustainable diets, behavioural intervention, policy framework

## Abstract

Background: Food waste is often framed as a technical or awareness problem, yet it persists most in societies best equipped to prevent it, pointing to overconsumption rather than logistics alone. This study tests whether food-system abundance, environmental pressure and food waste co-vary across countries, and whether food ethics and food citizenship help explain it. Methods: Using cross-country indicators from FAO, WHO, Global Carbon Project, World Bank, and UNEP for 20 purposively selected countries, a composite Systemic Pressure Index (five standardised indicators: dietary energy supply, obesity, meat consumption, CO_2_ emissions, food waste) yielded a three-tier typology, cross-checked with principal component analysis. Results: Tier 1 (US, Australia, Mexico, Romania, Greece, Brazil, Poland) shows the highest abundance and pressure. Food waste, however, loads on a distinct PCA dimension (PC2, 20.8% variance, r = 0.93 vs. r = 0.33 with PC1); the lowest-pressure tier splits into an affluent-moderate subtype (Sweden, Japan; among the lowest food-waste levels in the sample (with only the Netherlands, Tier 2, recording a lower figure)) and a lower-income/constrained subtype with comparatively high waste (125.6 kg/capita/year) despite minimal consumption. Conclusions: Food waste is comparatively weakly associated with the abundance dimension rather than behaving as a direct by-product of it. Food ethics and food citizenship offer an interpretative framework pointing toward differentiated, behavioural and policy interventions for food security and sustainability across typology tiers, rather than a demonstrated causal outcome.

## 1. Introduction

This research was originally presented at the RETASTE: Rethink Food Resources, Losses, and Waste 6th International Conference, Rethymno, Greece, 16–18 September 2026, with the abstract published as part of the conference materials [1].

Feeding a global population expected to approach nine or ten billion people, without breaching key environmental limits, is one of this century’s defining challenges, and food waste sits squarely inside it [2]. Policy documents and much of the scientific literature still tend to treat food waste mainly as a technical problem—losses along the supply chain, poor storage, or gaps in what consumers know about portioning, labelling and food handling [3]. That framing is not wrong, and it has produced genuinely useful fixes: better cold chains, clearer date labels, redistribution schemes. But it struggles with one stubborn fact. Food waste stays high precisely in the societies with the greatest technical capacity to prevent it. Early global assessments already put the scale of the problem at roughly one-third of food produced for human consumption [4], a baseline the Food Waste Index Report 2024 broadly confirms: about 1.05 billion tonnes of food were wasted worldwide in 2022—some 19% of what reaches consumers at retail, food-service and household level, on top of a further 13% lost earlier in the chain—while 733–783 million people went hungry that same year [5,6]. That coexistence of large-scale waste and persistent hunger is not a peripheral detail but arguably a central policy failure of the global food system in its own right, one that calls for tackling both problems together rather than as separate agendas [7]. Food loss and waste also account for an estimated 8–10% of global greenhouse gas emissions—roughly five times what aviation produces—and cost the global economy in the order of USD 1 trillion a year [6].

Efficiency alone cannot explain why abundance, waste and deprivation keep turning up together. A more plausible reading situates food waste inside a wider pattern of overconsumption: food systems that deliver far more dietary energy, animal-source protein and general material throughput than anyone needs, with an environmental footprint that grows to match [8,9]. Seen this way, waste is not a leak in an otherwise well-tuned system—it is one visible symptom of a food culture built around abundance rather than sufficiency. This study grows directly out of that argument and out of a related, single-country study that proposed food ethics as a fifth pillar of food security, alongside availability, access, utilisation and stability, using Romania as the case study [3]. What changes here is scope: instead of one country, 20, so that the abundance–waste–pressure pattern already documented nationally can be tested as a broader, cross-country regularity rather than a local peculiarity. The contribution pursued here is not the general observation that food waste is not confined to rich countries, which the UNEP Food Waste Index Report 2024 already establishes [6]; that pattern is a starting point, not a finding, of this study. The specific contribution is twofold: a multivariate test, via principal component analysis on five jointly standardised indicators, of whether food waste loads on the same dimension as caloric, meat, obesity and CO_2_ abundance or on a separate one, and an income-stratified subtype split within the lowest-pressure tier showing that the same low-waste outcome can sit on either side of a loss/waste distinction depending on income context, mapped onto differentiated policy instruments.

The aim of the study is to test, empirically and across a diverse set of countries, whether food-system abundance and food waste are part of the same phenomenon, and to interpret whatever pattern emerges through the lens of food ethics and food citizenship. This aim is pursued through three research questions.

- RQ1. Do countries with higher food-system abundance (dietary energy supply, obesity, animal-source food intensity) show higher environmental pressure (greenhouse gas emissions, land use) in this sample?
- RQ2. Does food waste scale with that same abundance dimension, or does it behave as a comparatively independent outcome—and, where it does not scale with abundance, does the underlying driver differ between higher- and lower-income contexts?
- RQ3. Can food ethics and food citizenship serve as a coherent interpretative framework linking these empirical patterns to behavioural responsibility, and can that framework be translated into differentiated, actionable governance instruments?

Answering these questions also speaks to the food security and food sustainability agenda: rather than one uniform food-waste policy, we ask whether behavioural, food-ethics-based interventions and a differentiated set of regulatory, fiscal and infrastructural policy instruments respond more accurately to how abundance, waste and pressure actually co-vary across income contexts. As a first indication of that contrast, Tier 1 countries average 148.0 kg/capita/year of food waste against 125.6 kg/capita/year for the lower-income/constrained subtype despite much lower abundance and pressure on every other indicator (Section 4.4 reports the full tier-by-tier comparison). Section 4 and Section 5 return explicitly to each question in turn. To answer them, a comparative typology of 20 countries spanning high-, middle- and lower-income contexts on five continents is built from indicators of dietary energy supply, obesity, animal-source food intensity, greenhouse gas emissions, land use and food waste, all drawn from Our World in Data (OWID) and the primary sources behind it—FAO, WHO, the Global Carbon Project, the World Bank and UNEP.

## 2. Theoretical Background

The conventional food security framework—availability, access, utilisation, stability—tells whether a food system can technically deliver adequate nutrition, but says almost nothing about whether it does so within environmental limits or in a way that is distributively fair. That framework traces back to an economic tradition that defined food security primarily in terms of entitlements and access rather than aggregate availability alone [10]. Clapp et al. [11] extend this conventional framework to six dimensions by adding agency and sustainability to availability, access, utilisation and stability; the five indicators used in the present study map most directly onto the availability, utilisation and stability dimensions, and do not themselves measure agency or sustainability, a scope limitation stated here rather than implied. Balan et al. [3] treat the framework’s silence on environmental limits and distributive fairness as consequential rather than incidental: a country can look food-secure by every conventional metric while wasting several million tonnes of food a year and carrying a heavy burden of diet-related disease, which is exactly what they document for Romania, where an estimated 3.4 million tonnes of food waste coexists with food insecurity affecting close to 15% of the population. Their response is to name the gap rather than leave it implicit, proposing food ethics—moderation, respect for resources, regard for environmental limits, awareness of the impact of animal-sourced food—as an explicit fifth pillar of food security.

A separate but related literature in agricultural and environmental ethics gives this idea a name: food citizenship, the everyday behavioural face of such ethical commitments. This behavioural face sits within a broader tradition of political consumerism, in which private consumption choices are treated as a legitimate arena for pursuing collective political and ethical goals rather than purely private ones [12], and within work on the geographies of ethical consumption that traces how such commitments travel from individual choice into wider systems of provision [13]. Wilkins [14] was among the first to give the food-specific version of this idea a name, defining food citizenship as food-related behaviour that supports rather than undermines a democratic, socially just and environmentally sustainable food system—the food citizen, in her framing, stands in contrast to the passive consumer whose choices track mainly price and convenience. De Tavernier [15] pushes the idea further by asking whether responsible consumption is actually a duty, arguing that food choices are a form of political engagement in which individuals weigh personal and environmental consequences together, with government acting as mediator between individual autonomy and collective welfare. Lozano-Cabedo and Gómez-Benito [16] go on to formalise the concept into an eight-proposition model, in which food citizenship combines an extended right to food with civic obligations exercised both through private consumption and through participation in food system governance. This behavioural literature is not naïve about how consistently such commitments translate into practice: consumers routinely handle environmentally and ethically loaded food risks with ambivalence rather than settled conviction, weighing competing everyday priorities against abstract principles [17].

Together, these strands suggest a two-step chain that this study takes as its interpretative scaffold: food ethics is the normative content—what moderation, resource-respect, environmental limits and animal-impact awareness actually require—and food citizenship is that content made visible, in aggregate consumption and waste patterns. The distinction is not just conceptual housekeeping; it generates a testable expectation. If food ethics is genuinely internalised at societal scale, it should leave a trace in macro-level indicators of dietary intensity, environmental pressure and waste—and that trace should not simply track income. Sustainable-diets research offers a further anchor here. The EAT-Lancet Commission [8] modelled planetary boundaries for food systems and found that diets in most high-income countries substantially overshoot sustainable levels of red meat and total caloric intake, while several lower- and middle-income countries sit below them. Subsequent work has reinforced and generalised that finding: diets that jointly track environmental sustainability and human health outcomes converge on broadly similar recommendations across very different national contexts [8,9,18], food-demand management is now recognised as a mitigation lever in its own right alongside supply-side efficiency gains [19], modelled pathways for keeping the food system within planetary boundaries depend heavily on demand-side change rather than technology alone [20], and reduced meat consumption specifically is identified as one of the more direct levers for easing biodiversity and environmental pressure [21]. If a comparable pattern holds once waste and emissions are considered jointly, that would support treating overconsumption, rather than scarcity or inefficiency on its own, as the more fundamental driver of the food waste problem this study is concerned with.

One more distinction, insisted on throughout the food loss and waste literature, is preserved here as well: losses further up the supply chain—at harvest, storage, processing, distribution—are structurally and causally different from waste further down, at retail, food service and household level [5,6,22,23]. The former tracks infrastructure, climate and market access; the latter tracks household behaviour, portioning habits and cultural attitudes toward food. A sociological account of household food waste warns against reducing this second category to simple carelessness or ignorance, situating it instead within the ordinary, often unnoticed rhythms of domestic food provisioning, cooking and eating [24]. A substantial review literature has since mapped the drivers behind consumer-level waste in more granular detail—behavioural, economic, infrastructural and normative factors interacting rather than any single cause dominating [25,26,27,28,29]—which is precisely the diversity of drivers this study’s typology is designed to sort by context rather than treat as uniform. A food-ethics framework, and whatever food-citizenship behaviour it might motivate, speaks mainly to the consumer-level category—which, notably, is also the part of the problem that has grown rather than shrunk as incomes have risen [5].

**Figure 1:**
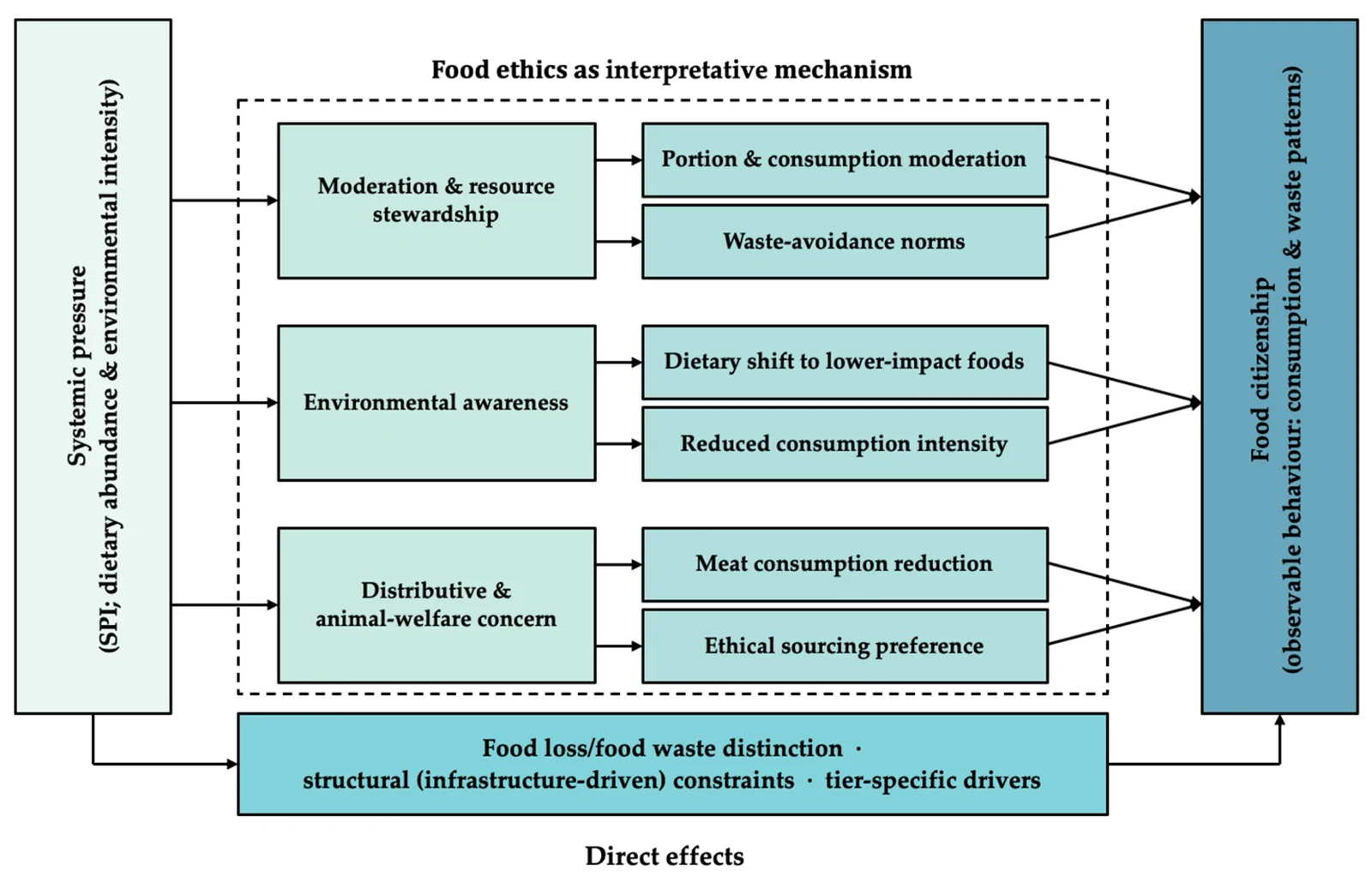
Theoretical framework linking systemic pressure to food citizenship through the interpretative mechanism of food ethics, with structural, infrastructure-driven loss shown as a direct effect.

The figure below summarises this interpretative scaffold (Figure 1).

Systemic pressure—the material condition captured empirically by the SPI and the resulting typology (Section 3.3 and Section 4)—does not translate into food-citizenship behaviour automatically. Food ethics is treated here as the mechanism connecting the two, operating through three broad channels drawn from the literature reviewed above: moderation and resource stewardship, environmental awareness, and distributive and animal-welfare concern, each plausibly shaping a distinct behavioural expression visible in aggregate consumption and waste data. Not all of the pressure–behaviour relationship is expected to run through this ethical channel, however: consistent with the loss/waste distinction just drawn, a substantial share of measured food waste—particularly in the lower-income/constrained subtype identified in Section 4.4—more plausibly reflects structural, infrastructure-driven loss than discretionary behaviour, and is shown here as a direct effect that bypasses the food-ethics mechanism. The framework is offered as an interpretative device for organising the empirical results reported in Section 4 and Section 5, not as a structural model estimated from the data.

## 3. Materials and Methods

### 3.1. Data Sources and Country Selection

Country-level indicators come from OWID, which aggregates and harmonises primary series from the FAO, the WHO Global Health Observatory, the Global Carbon Project, the World Bank’s World Development Indicators, and the UNEP Food Waste Index 2024 [5,30,31,32,33,34]. Twenty countries were selected deliberately and purposefully, not at random, against four explicit criteria chosen to serve the study’s aim. First, income diversity: countries were drawn from all four World Bank income groups—high-, upper-middle-, lower-middle- and low-income—so that the abundance–pressure relationship could be tested across the full income range rather than within a single bracket. Second, regional spread: selection aimed to cover as many world regions as the underlying data sources allowed with acceptable completeness, rather than clustering in one or two continents. Third, dietary and agricultural heterogeneity: the sample deliberately mixes large net agricultural exporters (United States, Australia, Brazil) with net importers, and high-meat-consumption diets with predominantly plant-based ones, so that the resulting typology does not simply relabel a single dietary pattern under different names. Fourth, data completeness: only countries with usable, reasonably recent values for all six indicators across every source were retained, which excluded a number of otherwise relevant candidates with incomplete OWID coverage. Romania and a set of Sub-Saharan African countries (Nigeria, Kenya, Ethiopia) were additionally prioritised for continuity with the authors’ own related single-region work on the land-use consequences of plant-based diets in Romania [35] and on protein-supply structure and sustainable food systems in Sub-Saharan Africa [36], so that this cross-country typology could be read alongside those more focused analyses rather than in isolation. The resulting sample—United States, Australia, Mexico, Romania, Greece, Brazil, Poland, Germany, Italy, United Kingdom, China, France, Netherlands, Sweden, Indonesia, Japan, Nigeria, India, Kenya and Ethiopia—covers North and South America, Western, Southern, Eastern and Northern Europe, East and South-East Asia, South Asia, Oceania, and West and East Africa, and spans all four income groups; it remains a purposive rather than a probability sample, so the typology it supports should be read as descriptive of these 20 cases rather than as statistically generalisable to a wider population.

### 3.2. Indicators

Six indicators were kept, matching the abundance and impact metrics identified at the study’s design stage:

- Daily dietary energy supply per capita (kcal/day, food balance sheets, most recent year available, 2018–2023 depending on country coverage, a food-balance-sheet proxy for dietary intake rather than a direct measure of it);
- Prevalence of obesity among adults (%, BMI ≥ 30, age-standardised adult prevalence—a multifactorial outcome not attributable to diet alone);
- Meat consumption per capita (kg/year, a supply-based proxy for intake rather than consumption itself), standing in for animal-source food intensity;
- Production-based CO_2_ emissions per capita (tonnes/year, a general proxy for environmental pressure rather than a food-system-specific or trade-adjusted footprint;
- Arable land per capita (hectares), a measure of land availability, not dietary footprint;
- Food waste per capita (kg/year, retail, food-service and household components combined), measured following the conventions set out under SDG Indicator 12.3.1 [37].

Sources, units and reference years appear alongside the results in Table 1; the mixed reference years are flagged as a limitation in Section 3.6 rather than smoothed over.

### 3.3. Composite Systemic Pressure Index and Typology

Getting from six separate indicators to the typology the study design called for meant building a composite: a Systemic Pressure Index (SPI), the unweighted mean of the standardised (z-score) values of five of the six indicators—caloric supply, obesity, meat consumption, CO_2_ emissions and food waste—oriented so that a higher value always means more pressure. Each indicator was first standardised across the 20-country sample using the usual z-score transformation,

(1) $$z_{i,c}=(x_{i,c}-{\overset{-}{x}}_{i})/s_{i}$$

where:

zi,c—the standardised (z-score) value of indicator i for country c;

xi,c—the raw, unstandardised value of that indicator for that country;

${\overset{-}{x}}_{i}$—the sample mean of indicator i across all 20 countries;

and

si—the population standard deviation of indicator i across the 20 countries.

The SPI for country c was then computed as the unweighted average of the five standardised indicators (caloric supply, obesity, meat consumption, CO_2_ emissions, food waste; k = 5),

SPI_c_ = (1/5) Σ_i=1_^5^ z_i,c_ (2)

where:

SPIc—the Systemic Pressure Index for country c;

zi,c—the standardised value of indicator i for country c, as defined in Equation (1);

and the summation runs over the five indicators, i = 1, …, 5 (caloric supply, obesity, meat consumption, CO_2_ emissions, food waste).

Both the standardisation step in Equation (1) and the aggregation step in Equation (2) follow standard composite-indicator construction practice, as codified in the OECD/Joint Research Centre handbook on constructing composite indicators [38], which the SPI draws on directly for its normalise-then-aggregate structure, while deliberately keeping the aggregation unweighted given the exploratory rather than confirmatory aim of the typology. Arable land per capita was left out of this pooled index on purpose and kept as a separate, contextual variable, since it reflects how much farmland a country has relative to its population at least as much as it reflects consumption behaviour; folding it in with the demand-side indicators would blur structural endowment together with behavioural pressure, a distinction the study design keeps explicit. Countries were then split into three tiers by SPI tercile—that is, by ranking the 20 countries’ SPI values and cutting the ranked list at the 33rd and 67th percentile: Tier 1 (“high-abundance, high-pressure”, top third), Tier 2 (“intermediate”, middle third), Tier 3 (“low measured pressure”, bottom third). Tier 3, though, turned out to bundle together countries with very different reasons for scoring low, so it was split again, this time on substantive, income-based grounds, into an “affluent-moderate” subtype (high-income countries with low pressure scores—Sweden and Japan) and a “lower-income/constrained” subtype (Indonesia, Nigeria, India, Kenya, Ethiopia). Section 4 returns to why that split matters.

#### Robustness Checks

Equal weighting across the five indicators, the inclusion of food waste in an index later used to help explain food-waste variation, and the mixed reference years of the underlying indicators are all substantive modelling choices rather than neutral defaults. Four checks were therefore run on the same 20-country dataset: an SPI variant excluding food waste; a leave-one-indicator-out series; an alternative-weighting variant that groups the three most strongly correlated “abundance” indicators (caloric supply, meat consumption, CO_2_ emissions) into a single component, averaged with obesity and food waste as three equal components; and a reference-year sensitivity check using alternative-vintage data where available. Full results are reported in Table 2; headline figures are summarised here.

Excluding food waste (four indicators: caloric supply, obesity, meat consumption, CO_2_ emissions) yields an SPI that correlates with the published five-indicator SPI at Spearman’s ρ = 0.9383 and reassigns four countries to an adjacent tier (Mexico and Brazil, Tier 1 → Tier 2; Germany and the United Kingdom, Tier 2 → Tier 1). Because this is identical to leaving food waste out in the leave-one-out series, it indicates the typology is directionally but not fully robust to including food waste. The tier-based narrative in Section 5 nonetheless uses the published five-indicator classification (Table 1) throughout, including for the four countries reassigned here (Mexico, Brazil, Germany, United Kingdom); the four-indicator variant is reported here strictly as a robustness check on the typology, not as an alternative basis for interpretation, so for these four countries specifically, the food-waste-related discussion in Section 5.1 carries a degree of circularity by construction. Leaving out each of the other four indicators in turn produces Spearman correlations with the published SPI ranging from ρ = 0.9805 (omitting caloric supply, no tier changes) to ρ = 0.9233 (omitting obesity, four tier changes: Romania and Poland, Tier 1 → Tier 2; Germany and China, Tier 2 → Tier 1); omitting meat consumption (ρ = 0.9910) or CO_2_ emissions (ρ = 0.9925) each reassigns only two countries.

Grouping caloric supply, meat consumption and CO_2_ emissions into a single “abundance” component, rather than averaging all five indicators directly, correlates with the published SPI at ρ = 0.9624 and reassigns four countries (France and the Netherlands, Tier 2 → Tier 3; Indonesia and Nigeria, Tier 3 → Tier 2).

A reference-year sensitivity check compared each country-indicator value against the closest available alternative-vintage estimate from the same source, restricted to plausible adjacent years and excluding any candidate match differing from the manuscript’s figure by more than 3% of the reported value—a relative rather than absolute threshold, since the four tested indicators differ by orders of magnitude in scale (kcal/day, kg/year, percentage points and tonnes/year), so a single absolute cutoff would not be comparable across them; a larger relative gap indicates a different data-source vintage rather than a genuine one-year shift, consistent with FAO’s food balance sheets being revised retrospectively. Of the 80 country-indicator cells tested (20 countries × caloric supply, meat consumption, obesity, CO_2_ emissions; food waste was not tested, since the UNEP Food Waste Index 2024 is a single-vintage source), 74 had a trustworthy alternative-year match; recomputing the SPI with these alternative values leaves every country’s tier assignment unchanged (Spearman’s ρ = 1.0000 against the published SPI), with no tier changes among the tested cells. This check is necessarily bounded by whatever alternative-vintage observations happen to exist in the OWID-compiled series for four of the five indicators (food waste, a single-vintage UNEP source, could not be tested), so it should be read as supportive of, rather than exhaustive evidence for, the typology’s robustness to reference-year choice.

None of these checks substitutes for an independent validation of the typology: the principal component analysis reported in Section 4.6 is computed on the same five standardised indicators as the SPI and is therefore best read as an internal, descriptive cross-check on the same data, not as independent confirmation. Bootstrap 95% confidence intervals for the pairwise correlations (Table 4) and a bootstrap stability check for the PCA explained-variance ratios and the food-waste loading (reported alongside Table 5) are discussed further in Section 4.6.

### 3.4. Correlation Coefficients

Bivariate associations reported throughout this study (for example, the r-values in Section 4 linking caloric supply, obesity, meat consumption and food waste) are Pearson product-moment correlation coefficients, computed for each pair of indicators x and y across the 20 countries as

(3) $$r_{xy}=[{\Sigma_{c=1}}^{20}(x_{c}-\overset{-}{x})(y_{c}-\overset{-}{y})]/[\surd ({\Sigma_{c=1}}^{20}{\left(x_{c}-\overset{-}{x}\right)}^{2})\;\surd ({\Sigma_{c=1}}^{20}{\left(y_{c}-\overset{-}{y}\right)}^{2})]$$

where:

r_xy_—the Pearson correlation coefficient between indicators x and y; xc and yc are the values of the two indicators for country c;

$\overset{-}{x}$ and $\overset{-}{y}$—their sample means across the 20 countries;

and both summations run over all 20 countries, c = 1, …, 20.

The coefficient follows Pearson’s original formulation of a general measure of linear association between two variables [39], and ranges from −1 (perfect inverse association) to +1 (perfect direct association), with values near 0 indicating little linear association—the basis for describing food waste as “comparatively weakly associated” with the dietary-abundance dimension in Section 4 and Section 5 (statistical orthogonality, as used later for the PCA components, is a property of the derived components themselves, not evidence of statistical or causal independence between the underlying variables). Given the small sample (n = 20) and the exploratory purpose of the study, these coefficients are reported descriptively, without formal significance testing, consistent with the treatment of the SPI and PCA results below.

### 3.5. Principal Component Analysis

Pairwise correlation only goes so far in testing whether food waste really belongs to the same underlying dimension as dietary abundance, or sits apart from it, so a principal component analysis (PCA) was also run on the five standardised indicators feeding the SPI—caloric supply, obesity, meat consumption, CO_2_ emissions, food waste [40]. PCA itself dates to Pearson’s original geometric formulation of fitting lines and planes of closest fit to a system of points [41] and to Hotelling’s later development of the method as an analysis of a complex of statistical variables into orthogonal components [42]; the treatment here follows the standard modern exposition of the technique [40]. PCA re-expresses each country’s five standardised indicator values as a set of orthogonal components, each a linear combination of the original standardised indicators,

PC_k,c_ = Σ_i=1_^5^ w_k,i_ z_i,c_ (4)

where:

PC_k,c_—country c’s score on principal component k;

w_k,i_—the loading (weight) of indicator i on component k—the entries reported in Table 5, obtained as the eigenvectors of the 5 × 5 correlation matrix of the standardised indicators, ranked by the size of their associated eigenvalues;

Z_i,c_—the standardised value of indicator i for country c, as defined in Equation (1);

and the sum runs over the five indicators, i = 1, …, 5.

The share of total variance explained by a given component, reported in Section 4 (63.3% for PC1, 20.8% for PC2), is

EVR_k_ = λ_k_/Σ_j=1_^5^ λ_j_ (5)

where:

EVR_k_—the proportion of total variance explained by component k;

λ_k_—the eigenvalue associated with component k;

and the denominator sums the eigenvalues of all five components, j = 1, …, 5.

Each indicator’s correlation with a given component (also reported in Table 5) is computed with Equation (3), substituting each country’s score on that component for one of the two variables. Given the exploratory aim of the study and the modest sample (n = 20), PCA is used here to reduce dimensionality and describe pattern, not to run formal hypothesis tests or to replace the composite index.

### 3.6. Limitations

A few limitations bear directly on how the results should be read. Reference years differ across indicators (2018–2024) simply because each source updates on its own schedule, so the typology is best read as an approximate recent cross-section, not one synchronous snapshot. CO_2_ emissions per capita are production-based national totals rather than food-system-specific or trade-adjusted, consumption-based figures—a general proxy for environmental pressure, not a precise dietary carbon footprint. Arable land per capita captures cropland only, not pasture or total agricultural land, so it is a partial land-use proxy at best. The food waste figures come from national estimates of uneven methodological maturity—the UNEP index itself flags varying confidence levels by country—which is reason for caution specifically around cross-country comparisons of that indicator. The index measures retail, food-service and household-stage waste only; it does not capture upstream, pre-retail food loss. Section 5.1’s explanation of the lower-income/constrained subtype’s comparatively high per-capita figure by appeal to post-harvest and cold-chain constraints is accordingly presented there as an explicitly hedged hypothesis about likely downstream consequences of upstream constraints, not as a direct reading of an upstream-loss indicator this study does not have. The analysis also does not incorporate agri-food market prices or trade-flow indicators directly; where the governance instruments discussed in Section 5.2 rely on market-based mechanisms (metered pricing, fiscal incentives), their price-transmission effects merit dedicated market-level analysis in future work. Most importantly, the composite index and the tiers built from it are descriptive and exploratory: they establish an association between abundance-related indicators and environmental or waste pressure, not a causal claim, and the sample, deliberately diverse as it is, is not a probability sample and cannot support population-level statistical inference. Measurement uncertainty also varies systematically across countries and indicators. Supplementary Table S1 documents, for every country and indicator, the exact reference year, source vintage and, for food waste specifically, the UNEP Food Waste Index 2024’s own household-estimate confidence classification (High for the United States, Australia, the United Kingdom and Japan; Medium for China, India, Indonesia, Mexico, Brazil, Nigeria, Kenya and Ethiopia; Low for Romania; reported via Eurostat rather than UNEP’s own scale for Greece, Poland, Germany, Italy, France, the Netherlands and Sweden). This classification covers the household component only: the food-waste figure used in Table 1 is UNEP’s combined household, retail and food-service estimate, and for countries without their own retail/food-service study, that component is extrapolated at Low confidence (high-income countries) or Very Low confidence (upper-middle/lower-middle/low-income countries, “a very rough global average”). Indonesia, Nigeria, India, Kenya and Ethiopia all fall in the latter group, so their non-household components likely carry materially lower confidence than their Medium household rating—a reason for caution in both directions when interpreting their food-waste figures, not only a reason to expect undercounting.

### 3.7. Use of Generative Artificial Intelligence

During the preparation of this manuscript, the authors used the Research Assistant tool from Web of Science for assistance with the specialised literature search, Claude Sonnet 5 to improve wording, and the Grammarly app (v.1.2.300.1965) for text-editing correction. The authors have reviewed and edited the output and take full responsibility for the content of this publication.

## 4. Results

The results obtained provide a coherent empirical basis for the typology proposed in this study, linking each stage of the analysis to a verifiable computational step. Table 1 reports the full dataset, the resulting SPI, and the tier classification for all 20 countries.

The subsections that follow reproduce, in sequence, the intermediate computations underlying this index (the standardised indicator values, the pairwise correlation structure, and the principal component solution) with each table linked to the specific equation in Section 3 from which its values were derived, so that the full computational path from raw indicators to composite index and typology remains traceable and independently verifiable.

### 4.1. Cross-Country Typology and the Three-Tier Gradient

**Figure 2:**
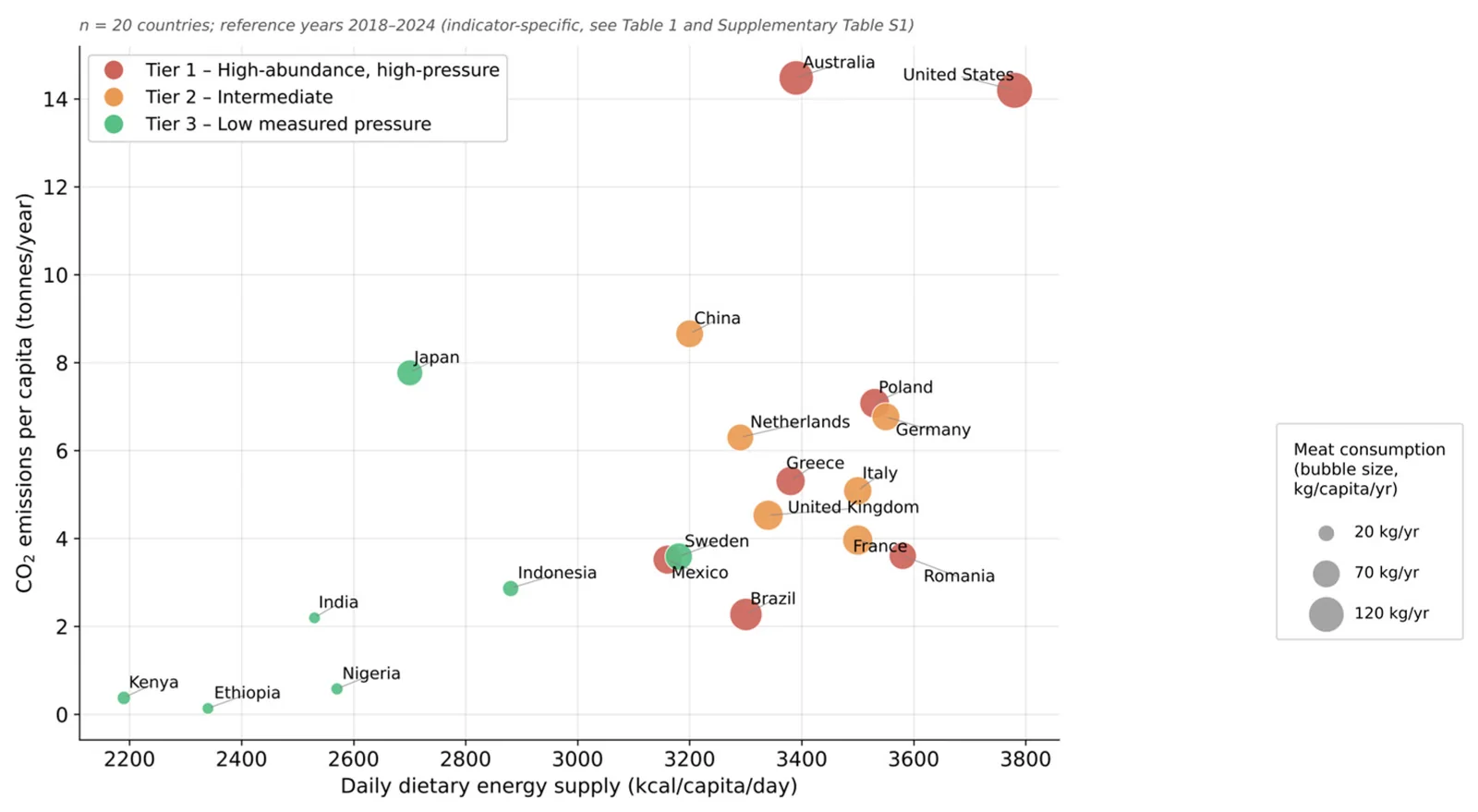
Daily dietary energy supply (kcal/capita/day) versus CO_2_ emissions per capita (tonnes/year). Bubble size is proportional to meat consumption per capita (kg/year); colour denotes systemic-pressure tier (Tier 1, Tier 2, Tier 3, as defined in Table 1). n = 20 countries; reference years 2018 –2024, indicator-specific (see Table 1 and Table S1).

Tier 1—United States, Australia, Mexico, Romania, Greece, Brazil, Poland (seven countries)—tops all other tiers on every headline measure: the highest mean caloric supply (3446 kcal/day), the highest mean obesity prevalence (34.7%), the highest mean meat consumption (91.4 kg/year), and the highest mean food waste (148.0 kg/capita/year). Tier 2 (Germany, Italy, United Kingdom, China, France, Netherlands; six countries) sits in between on nearly every indicator. Tier 3 (Sweden, Indonesia, Japan, Nigeria, India, Kenya, Ethiopia; seven countries) has the lowest mean SPI (−0.8), driven mainly by much lower caloric supply (2627 kcal/day) and meat consumption (25.6 kg/year). Mean food waste does not track this gradient monotonically: it falls from 148.0 kg/capita/year in Tier 1 to 102.0 kg/capita/year in Tier 2, then rises again to 112.6 kg/capita/year in Tier 3, a pattern examined further in Section 4.4. Across the full sample, SPI values span a range of exactly 3.0 index points, from the United States (SPI = 1.67, the highest) to Ethiopia (SPI = −1.33, the lowest)—a spread wide enough to place genuinely different food systems side by side, which was the point of the purposive sampling strategy set out in Section 3.1. This three-tier gradient matches what the study design anticipated: countries with more abundant, more resource-intensive food systems carry correspondingly higher environmental pressure (Figure 2).

As a first robustness check on the typology, Table 2 reports how the SPI and tier assignments respond to excluding food waste, to leaving out each indicator in turn, to an alternative weighting, and to a reference-year sensitivity check; methodology is given in Section Robustness Checks.

### 4.2. Standardised Indicators

Table 3 reports the intermediate step behind Table 1’s SPI column: the z-score standardisation of the five pressure indicators for each of the 20 countries, computed with Equation (1).

Presenting these values directly, rather than only the aggregated SPI, makes clear how each country’s pressure score is built up indicator by indicator, and lets the pattern already visible in Table 1 be traced back to its components: the United States, Australia and Mexico post the largest positive z-scores on food waste specifically (1.01, 1.51 and 2.55), while Ethiopia and Kenya post the most negative z-scores across nearly every indicator, consistent with the low end of the Tier 1–Tier 3 gradient described in Section 4.1.

### 4.3. Bivariate Correlations Among the Pressure Indicators

Table 4 reports the full set of pairwise Pearson correlations among the five indicators feeding the SPI, computed via Equation (3), together with bootstrap 95% confidence intervals (2000 resamples, percentile method).

Four of the five (caloric supply, obesity, meat consumption and CO_2_ emissions) correlate moderately to strongly with one another, from r = 0.46 (obesity with CO_2_ emissions) to r = 0.87 (caloric supply with meat consumption), consistent with a single underlying dimension of dietary and environmental abundance. Food waste stands apart from that cluster: its correlations with the other four indicators are consistently weaker and more uneven, ranging from r = 0.05 with caloric supply to r = 0.43 with obesity. That last figure is worth flagging in its own right—food waste correlates more closely with obesity than with the caloric-supply variable it might be expected to track most directly—an early sign, confirmed by the PCA in Section 4.6, that food waste responds to a partly distinct set of drivers rather than simply scaling with how much a population eats.

### 4.4. The Tier 3 Split—Affluent-Moderate Versus Lower-Income/Constrained

Splitting Tier 3 into subtypes shows that the aggregate gradient is hiding two quite different stories. The affluent-moderate subtype (Sweden, Japan) is high-income and food-secure yet has markedly lower caloric supply (2940 kcal/day on average), lower obesity (10.6%), and—the striking part—among the lowest food waste of any group in the sample, at 80.3 kg/capita/year (only the Netherlands, in Tier 2, records a lower per-capita figure, at 62 kg/capita/year). The lower-income/constrained subtype (Indonesia, Nigeria, India, Kenya, Ethiopia) goes further still on caloric supply (2502 kcal/day) and meat consumption (10.2 kg/year), yet its mean food waste, 125.6 kg/capita/year, comes in over 50% higher than the affluent-moderate subtype’s and close to the Tier 1 average. That fits the broader correlation pattern reported in Section 4.3: dietary energy supply, obesity and meat intake move together fairly tightly—one underlying the “dietary abundance” dimension, but food waste refuses to scale with it, consistent with related evidence that household food waste in Europe tracks national economic conditions in ways that do not reduce neatly to overall consumption abundance [43]. Some of the lowest-consumption countries in the sample post relatively high per-capita waste; two of the more moderate, high-income countries post the lowest waste of all.

### 4.5. Land-Use Pressure

Land-use pressure, kept separate as the methodology specifies, tells a messier story. Australia and the United States pair high caloric supply with by far the largest arable land per capita (1.24 and 0.48 ha), which fits their land-abundant agricultural economies, but several other Tier 1 countries reach comparably high caloric supply on much smaller land endowments—Greece at 0.20 ha, Romania at 0.45 ha. High dietary abundance, in other words, is not simply a function of how much land a country has; trade, intensification and dietary composition can get you there just as well. For Romania specifically, related modelling of the land-use consequences of a shift toward plant-based diets suggests that even this relatively small arable-land footprint still leaves meaningful room for further land-use reduction through dietary change alone [35].

### 4.6. PCA Confirmation of the Abundance–Waste Separation

The PCA puts a number on this last point, Because it is computed on the same five standardised indicators as the SPI, it functions here as an internal, descriptive cross-check on the same data rather than an independent validation of the typology (Section Robustness Checks). computed via Equations (4) and (5) (Table 5).

Its first component (PC1) accounts for 63.3% of total variance across the five indicators, and correlates strongly with caloric supply (r = 0.90), meat consumption (r = 0.95), obesity (r = 0.86) and CO_2_ emissions (r = 0.78)—confirmation that these four really do move together as one dominant “dietary abundance and environmental intensity” dimension, consistent with evidence that diet-related environmental impacts scale together across producers and consumers alike [9]. Food waste barely correlates with PC1 (r = 0.33); instead, it loads almost entirely onto the second component (PC2, r = 0.93), which picks up a further 20.8% of variance, for 84.1% cumulative across the first two components, both computed via Equations (4) and (5). That is the multivariate version of the bivariate pattern already visible in Table 4: food waste is comparatively weakly associated with the dietary-abundance dimension organising the other four indicators, rather than trailing behind it as a downstream effect.

**Figure 3:**
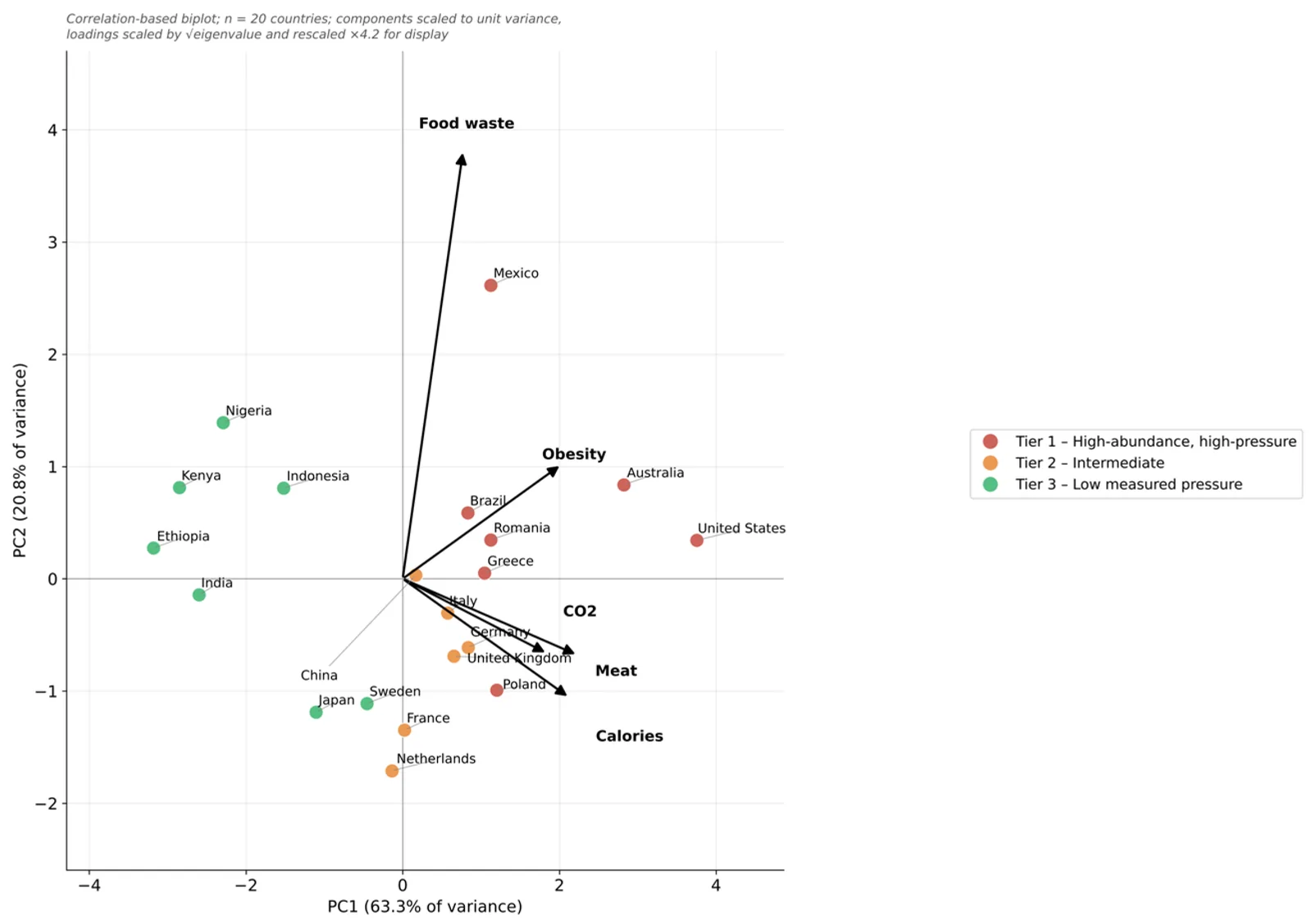
Correlation-based PCA biplot of the five standardised food-system pressure indicators (n = 20 countries); components are scaled to unit variance, and loadings are scaled by the square root of their eigenvalue, then rescaled for display. Arrows show indicator loadings; PC1 (63.3% of variance) represents dietary abundance/environmental intensity, PC2 (20.8% of variance) is dominated almost exclusively by food waste. Points are coloured by systemic-pressure tier, as in Figure 2; reference years 2018–2024, indicator-specific (see Table 1 and Table S1).

Figure 3 plots all 20 countries in PC1–PC2 space alongside the indicator loading vectors. Tier 1 countries cluster high on PC1 (high abundance/intensity) but scatter on PC2—Mexico and Australia sit noticeably higher, meaning relatively more waste, than Poland or Greece—while Tier 3b countries cluster low on PC1 but spread across a wide range of PC2 values, which is essentially a picture of the subtype split described in Section 4.4: Nigeria, despite low abundance, still registers comparatively high relative waste (positive PC2), while India and Ethiopia do not.

Table 6 completes the picture by reporting each country’s own score on the first two components, computed via Equation (4) and plotted in Figure 3. Positive PC1 values indicate higher dietary abundance and environmental intensity; positive PC2 values indicate higher relative food waste. The United States (PC1 = 3.75) and Ethiopia (PC1 = −3.18) anchor the two ends of the abundance dimension, while Mexico’s markedly positive PC2 score (2.62)—the highest in the sample—confirms it as the clearest case of high relative food waste once the dietary-abundance dimension is held constant.

## 5. Discussion

This section interprets the results just presented against the food-ethics and food-citizenship framework set out in Section 2, and then translates the resulting typology into governance implications. Section 5.1 returns directly to the three research questions and situates the Tier 1–Tier 3 gradient, and the Tier 3 split in particular, within that interpretative framework. Section 5.2 then asks what the typology implies for policy design and proposes a differentiated set of governance instruments matched to the driver identified in each typology cell.

### 5.1. Food Ethics as Interpretative Framework, Food Citizenship as Behavioural Expression

Tier assignments refer to the published five-indicator Systemic Pressure Index (Table 1). The four-indicator variant introduced in Section Robustness Checks serves solely as a robustness check on this classification and is not used as an alternative basis for interpretation here. For the four countries it reassigns (Mexico, Brazil, Germany, and the United Kingdom), the food-waste-related interpretations presented below should accordingly be read in conjunction with the circularity caveat noted in Section Robustness Checks.

The results in Section 4 answer the three research questions set out in the Introduction directly. On RQ1, yes: countries with higher food-system abundance show higher environmental pressure in this sample, visible both in the Tier 1–Tier 3 gradient (Table 1) and in the first principal component, which explains 63.3% of variance and correlates strongly with caloric supply, meat consumption, obesity and CO_2_ emissions alike. On RQ2, no: food waste does not scale with that same abundance dimension. It correlates only weakly with PC1 (r = 0.33) and loads instead onto a largely separate second component (PC2, r = 0.93), and the driver behind it clearly differs by income context—plausibly discretionary and behavioural in the affluent-moderate subtype (Sweden, Japan), plausibly structural and infrastructure-driven in the lower-income/constrained subtype (Indonesia, Nigeria, India, Kenya, Ethiopia), despite both showing low consumption. On RQ3, the remainder of this section argues that food ethics and food citizenship do provide a coherent interpretative framework for this pattern, and one that translates into concrete, differentiated governance instruments (Section 5.2) rather than staying at the level of normative aspiration, though, as the final part of this section makes clear, this last claim rests on suggestive rather than confirmatory evidence.

The Tier 1 pattern (high caloric supply, high obesity, high meat intake, high emissions and high waste, all moving together) matches the structural gap between material abundance and responsible consumption flagged at the outset of this study. Across national contexts as different as a Western agricultural exporter (United States, Australia), a Central European economy (Romania, Poland) and a Southern European Mediterranean-diet country (Greece), crossing a certain threshold of dietary abundance seems to bring elevated environmental pressure and waste along with it, regardless of region or agricultural endowment. Read through the food-ethics lens Balan et al. [3] propose, this looks like the empirical signature of moderation, or its absence, operating at societal scale: none of the four ethical dimensions they name—moderation, respect for resources, environmental limits, animal-impact awareness—is a question of technical capacity in any of these countries, which all have well-developed food infrastructure. What differs is whether consumption norms have internalised any of the four.

The Tier 3 subtype comparison is, if anything, the most theoretically interesting result here. Sweden and Japan achieve low systemic pressure and, notably, among the lowest food waste in the sample (only the Netherlands, Tier 2, records a lower figure), while remaining high-income, food-secure societies with full access to modern food infrastructure—so their low pressure cannot be chalked up to constrained availability, unlike Ethiopia, Kenya, Nigeria or India, where lower caloric supply and meat consumption more plausibly reflect purchasing power and access constraints than any chosen moderation. That is exactly the distinction food citizenship, as developed by Wilkins [14], De Tavernier [15] and Lozano-Cabedo and Gómez-Benito [16], was built to capture: behaviour that supports a just and sustainable food system by choice, under conditions where less sustainable choices remain freely available. Sweden and Japan’s consumption profile—adequate but not maximal calories, comparatively moderate meat intake, and markedly low waste despite affluence—is consistent with food citizenship operating as the behavioural face of internalised food ethics at national scale, though it is not proof of it. Cultural dietary tradition, relative food prices, differences in food composition, and measurement differences across national waste-accounting systems are alternative explanations this study cannot rule out with national-level indicators alone, and are noted here as such. The lower-income/constrained subtype makes the opposite point: it shows why the food-ethics framework has to be applied with care, since low material throughput without choice is not evidence of moderation, and reading it that way would mean moralising what is really structural food insecurity. This concern is not hypothetical: related work on protein-supply structure in Sub-Saharan African food systems documents exactly this kind of structurally constrained consumption, where low intake reflects supply and affordability limits rather than restraint, reinforcing the need to keep the two readings apart [36].

The same comparison is also consistent with, though it cannot on its own establish, the distinction between supply-chain losses and consumer-level waste that both this study and the wider literature insist on keeping separate [5,6]. The UNEP Food Waste Index measures retail, food-service and household-stage waste only; it does not itself capture upstream, pre-retail food loss, so what follows is offered as a hedged hypothesis about likely downstream consequences of upstream constraints, not as a direct reading of an upstream-loss indicator this study does not have (Section 3.6). The lower-income/constrained countries here pair low consumption with comparatively high per-capita waste (125.6 kg/year, close to the Tier 1 mean of 148.0 kg/year)—a combination more consistent with upstream, infrastructure-driven loss (inadequate storage, cold chain, market access) than with discretionary household waste, a pattern independently documented for each of these countries or their region: Sub-Saharan Africa generally and Kenya specifically [44], Nigeria [45], Ethiopia [46], India, where cold storage is a necessary but not independently sufficient condition for loss reduction [47], and Indonesia [48]. The affluent-moderate subtype’s low waste (80.3 kg/year), by contrast, shows up where infrastructure is not the binding constraint, which makes a behavioural or normative explanation much more plausible. If that reading holds, it carries a direct policy implication: the same headline food-waste number can call for opposite interventions depending on which side of the loss/waste line it falls on—infrastructure and cold-chain investment where losses are structural, food-ethics-oriented behavioural and educational measures, capable of cultivating food citizenship, where waste is discretionary. That lines up with56 how the European Green Deal’s Farm to Fork Strategy [49] pairs supply-chain efficiency measures with consumer-behaviour targets.

The weak overall correlation between food waste and dietary abundance (r = 0.05) deserves to be underlined on its own terms. It means that, contrary to a simple “rich countries waste more” story, food waste is only weakly linearly associated with the abundance dimension, more responsive to norms, infrastructure and food culture than to income or caloric supply as such—not a passive by-product of affluence, a reading that lines up with evidence that household food waste relates to national economic conditions in a more complicated way than aggregate abundance alone would predict [43]. That weak association is what makes food ethics a necessary part of any credible waste-reduction strategy, not just a nice complement to efficiency measures. If waste tracked abundance mechanically, cutting abundance would be enough; because it does not, changing the normative content of food-related behaviour—cultivating food citizenship—has to stand as its own policy lever, which is exactly what Balan et al. [3] argue in proposing food ethics as an explicit fifth pillar of food security.

These findings should be interpreted in light of the study’s explicit scope conditions, set out in Section 3.6: the sample was purposively selected rather than drawn as a probability sample, the composite index is an unweighted average rather than a validated psychometric scale, and the cross-sectional design describes association rather than establishing causal direction. Sweden and Japan’s comparatively low obesity and meat consumption, for instance, plausibly reflect a combination of cultural dietary tradition and the moderation associated with food citizenship, and disentangling the two is a natural next step for the research programme this study opens up. Extending the country sample into a larger, systematically drawn panel, complementing the aggregate indicators with direct survey measures of food-citizenship attitudes and behaviour, and tracking the same countries over time would further strengthen and refine the typology identified here.

### 5.2. Governance and Policy Implications— Differentiated Instruments Across Typology Tiers

The typology → instrument mapping offered below is a plausibility argument, drawn from the typology’s own logic and from independently documented national case studies, not a causal evaluation of these instruments’ effects; none of the real-world examples cited here are offered as evidence that an instrument works because it was applied in that tier. Translating a typology into policy is really the point of Section 4’s results, so this subsection spells out what they imply for governance design, informed by the broader recognition that land use and food systems are now treated as integral parts of climate governance rather than a separate domain [50], and by international calls for context-specific, target-and-measure governance rather than one uniform global instrument [51]. Four instrument families already exist in national practice, and they map onto the four typology cells identified here with reasonable precision—which suggests that a differentiated governance mix, rather than one uniform policy, is both feasible and, on this evidence, better matched to the underlying driver of waste in each context.

Tier 1 countries combine retail- and household-level over-provision with genuine material abundance, and France offers a tested regulatory template for the retail end: its 2016 law against food waste (“Loi Garot”) bars large supermarkets from deliberately spoiling or destroying unsold edible food and requires donation agreements with charities instead—closing off the cheapest way of handling surplus exactly where abundance is highest [52]. Once that option is foreclosed, much of Tier 1’s remaining waste plausibly shifts to the household, where South Korea’s volume-based food waste fee is the best-documented behavioural instrument around: metered, RFID-enabled bins charge residents by the weight of what they throw away, turning a previously invisible cost into something visible and individually attributable, and the mechanism is credited with lifting the country’s food waste recycling rate from roughly 2% in 1995 to about 95% today [53]. This period also saw substantial parallel investment in separate-collection infrastructure and public information campaigns, so the fee alone cannot be credited with the full increase; the cited figure is also a recycling-diversion rate, not a waste-prevention (generation-reduction) rate, and is not treated here as evidence that the fee reduced how much food was wasted in the first place. Because it prices the act of wasting rather than merely urging moderation, this instrument operationalises food citizenship directly—it converts an ethical disposition into an everyday economic choice.

There is also a market and trade dimension to moderation-driven demand reduction in Tier 1 countries, and it is not incidental to food citizenship but part of its systemic logic. FAO’s State of Food and Agriculture 2019 report points out that food loss and waste reduction, if large enough, transmits through the supply chain and moves prices both upstream and downstream, with the direction and size of the effect depending on where in the chain it originates [54]. Applied to this typology: sustained consumer-level moderation in the more import-exposed, high-abundance segments of Tier 1 diets—the feed-grain and animal-protein imports underlying part of the meat consumption recorded here for Mexico and Greece, for instance—would be expected to ease derived import demand and reduce exposure to global price swings. In the sample’s large net agricultural exporters (United States, Australia, Brazil), the same demand reduction would more plausibly free up exportable surplus or ease domestic land-use pressure than cut import dependency, since import dependency was never really the issue there. The heterogeneity is worth flagging precisely because “reducing demand” is not one uniform market intervention—its trade and price effects hinge on each country’s specific position in agricultural trade. That is also why the governance instruments proposed here stay at the household/behavioural level rather than reaching for a blanket market intervention, leaving the aggregate trade and price consequences as an open empirical question rather than an assumed benefit.

Tier 2 countries have abundance too, just less extreme, and a more mixed loss/waste split, which points toward fiscal rather than prohibitive instruments. Italy’s Gadda Law (Law No. 166/2016) reduces the municipal waste tax owed by businesses that donate surplus food and cuts the administrative burden of doing so, nudging behaviour through cost incentives rather than bans, in pursuit of an explicit national target of halving food loss and waste by 2030 [55]. Informational instruments extend the same logic to the point of consumer choice—European date-labelling reform, clarifying “best before” against “use by”, and environmental or ethical labelling schemes modelled on nutrition labels—and both fit with the European Commission’s Farm to Fork Strategy, which pairs supply-chain targets with consumer-information measures across the EU countries well represented in this tier (Germany, Italy, France, Netherlands) [49]. Recent EU-wide monitoring, however, confirms that progress on food waste reduction remains uneven across member states, reinforcing the case for the country- and tier-specific instruments proposed here rather than a single EU-wide benchmark [56].

The affluent-moderate subtype of Tier 3—Sweden, Japan—poses a different kind of problem entirely: these countries already have the outcome, low food waste alongside full food security, that instruments elsewhere are trying to induce. The governance task is not correction but consolidation—keeping existing dietary and waste-minimising norms in place through public education, and treating these two cases as benchmarks for designing behavioural interventions elsewhere, rather than as targets for new regulation of their own.

For the lower-income/constrained subtype of Tier 3 (Indonesia, Nigeria, India, Kenya, Ethiopia), Section 4 shows comparatively high per-capita waste alongside low consumption, a combination more consistent with upstream, infrastructure-driven loss than with discretionary household behaviour, following the same hedged reading set out in Section 5.1. Consumer-facing instruments of the sort suited to Tier 1 and Tier 2 are a poor fit here; the governance priority is investment in post-harvest storage, cold chain and market access—the territory FAO and the development banks already work in—guided by internationally harmonised measurement frameworks such as SDG Indicator 12.3.1 so that progress can be tracked comparably across countries [37]—rather than behavioural or fiscal nudges aimed at households that are not really where the loss is coming from. Running a food-ethics-branded behavioural campaign here would repeat exactly the moralising error flagged in Section 5.1, treating structurally constrained consumption as if it were a failure to internalise ethics.

Table 7 lays out this differentiated governance mix. It is a first-order mapping drawn from the typology’s own logic, not a validated policy evaluation, and every cell would need context-specific adaptation. The underlying point is simple: one global “food waste policy” is unlikely to fit all four cells equally well, and the typology developed here offers one principled basis for deciding which instrument family to prioritise, and where.

## 6. Conclusions

This study set out to test, across a diverse sample of 20 countries, whether the coexistence of food abundance, environmental pressure and food waste already documented nationally for Romania [3] also holds as a broader, cross-country pattern, and to interpret whatever it found through the combined lens of food ethics and food citizenship. The cross-country typology built from OWID indicators shows a clear three-tier gradient: higher dietary abundance goes with higher obesity, higher meat consumption and higher emissions, though food waste itself does not track this gradient monotonically across tiers (148.0 → 102.0 → 112.6 kg/capita/year for Tiers 1–3; Section 4.1). Splitting the lowest-pressure tier apart, though, shows that low systemic pressure is not one single phenomenon. An affluent-moderate subtype, Sweden and Japan, achieves among the lowest food waste in the sample (only the Netherlands, Tier 2, records a lower figure) despite full food security), plausibly reflecting internalised moderation consistent with food citizenship. A lower-income/constrained subtype achieves low consumption alongside comparatively high waste, which more plausibly reflects structural, supply-side loss than any behavioural restraint. And food waste itself correlates only weakly with dietary abundance across the sample as a whole—consistent with it being a largely separate outcome rather than a mechanical by-product of affluence.

Together, these results are consistent with treating food ethics—moderation, respect for resources, regard for environmental limits, awareness of animal impacts—as the interpretative framework connecting material outcomes to behavioural responsibility, with food citizenship as its observable face in aggregate consumption and waste patterns. Practically, this means food waste prevention strategies should differ by context: infrastructure and supply-chain investment where losses are structural, food-ethics-oriented behavioural and educational measures where waste is discretionary and food citizenship has room to take hold. Long-term reduction of food waste, on this reading, depends not only on continued efficiency gains but on value-oriented behavioural change, a claim this study has tried to substantiate empirically rather than simply assert.

## Figures and Tables

**Table 1:** Cross-country indicators, Systemic Pressure Index (SPI, computed via Equations (1) and (2)) and typology (Tier 1 = high-abundance/high-pressure; Tier 2 = intermediate; Tier 3a = low-pressure/affluent-moderate; Tier 3b = low-pressure/lower-income-constrained). Sources: FAO, WHO, Global Carbon Project, World Bank and UNEP Food Waste Index 2024, compiled via OWID [5,30,31,32,33,34].

Country	Region	Kcal/cap/day	Obesity %	Meat kg/yr	CO_2_ t/cap	Arable ha/cap	Food Waste kg/cap/yr	SPI	Tier
United States	N. America	3780	42.9	122.8	14.2	0.48	158.6	1.67	Tier 1
Australia	Oceania	3390	31.8	112.34	14.48	1.24	176.9	1.37	Tier 1
Mexico	N. America	3160	36.1	77.52	3.52	0.19	214.7	0.77	Tier 1
Romania	E. Europe	3580	38.2	67.89	3.61	0.45	130	0.47	Tier 1
Greece	S. Europe	3380	33.7	78.92	5.31	0.2	122.1	0.42	Tier 1
Brazil	S. America	3300	28.8	98.79	2.28	0.27	144.5	0.38	Tier 1
Poland	E. Europe	3530	31.4	81.62	7.08	0.29	89	0.37	Tier 1
Germany	W. Europe	3550	24.2	71.32	6.77	0.14	108	0.29	Tier 2
Italy	S. Europe	3500	21.6	73.58	5.09	0.11	119	0.21	Tier 2
United Kingdom	W. Europe	3340	28.7	84.08	4.53	0.09	95.2	0.16	Tier 2
China	E. Asia	3200	8.2	70.57	8.66	0.09	141.7	0.15	Tier 2
France	W. Europe	3500	10.9	84.6	3.97	0.27	86	−0.14	Tier 2
Netherlands	W. Europe	3290	16.9	65.09	6.3	0.06	62	−0.26	Tier 2
Sweden	N. Europe	3180	16.4	68.06	3.59	0.25	80	−0.34	Tier 3a
Indonesia	SE Asia	2880	11.5	19.16	2.87	0.1	143.5	−0.53	Tier 3b
Japan	E. Asia	2700	4.9	60.42	7.77	0.03	80.6	−0.57	Tier 3a
Nigeria	W. Africa	2570	10.8	7.61	0.58	0.17	154.3	−0.81	Tier 3b
India	S. Asia	2530	7.2	6.63	2.2	0.12	97.3	−1.13	Tier 3b
Kenya	E. Africa	2190	11	10.95	0.38	0.11	122.6	−1.15	Tier 3b
Ethiopia	E. Africa	2340	2.4	6.58	0.14	0.15	110.1	−1.33	Tier 3b

**Table 2:** SPI and typology robustness checks: sensitivity of the published five-indicator typology to indicator inclusion, weighting and reference year (n = 20 countries).

Variant	Spearman’s ρ vs. Published SPI	Countries Reassigned	Reassignments (Tier Change)
Without food waste (4 indicators)	0.9383	4	Mexico T1→T2; Brazil T1→T2; Germany T2→T1; United Kingdom T2→T1
Leave out caloric supply	0.9805	0	None
Leave out obesity	0.9233	4	Romania T1→T2; Poland T1→T2; Germany T2→T1; China T2→T1
Leave out meat consumption	0.9910	2	Brazil T1→T2; Germany T2→T1
Leave out CO_2_ emissions	0.9925	2	Netherlands T2→T3; Sweden T3→T2
Alternative weighting (abundance block + obesity + waste)	0.9624	4	France T2→T3; Netherlands T2→T3; Indonesia T3→T2; Nigeria T3→T2
Reference-year sensitivity (74/80 trustworthy cells)	1.0000	0	None

**Note:** the leave-one-out “food waste” row is identical to the “without food waste” variant above and is omitted to avoid duplication. Reference-year sensitivity evaluated 80 country-indicator cells (20 countries × caloric supply, meat consumption, obesity, CO_2_ emissions); 6 were excluded as untrustworthy vintage matches (differing from the manuscript’s reported figure by more than 3% in relative terms—a relative rather than absolute threshold, since the four tested indicators span very different measurement scales, from kcal/day to percentage points to tonnes/year), concentrated in obesity prevalence (5 of 20 countries); the Spearman correlation and zero tier changes shown are for the 74 trustworthy cells. Food waste was not tested for reference-year sensitivity, since the UNEP Food Waste Index 2024 is a single-vintage source. See Section Robustness Checks for methodology.

**Table 3:** Standardised (z-score) values of the five pressure indicators (n = 20 countries).

Country	Caloric Supply (z)	Obesity (z)	Meat (z)	CO_2_ (z)	Food Waste (z)
United States	1.44	1.82	1.74	2.35	1.01
Australia	0.56	0.90	1.43	2.42	1.51
Mexico	0.04	1.26	0.41	−0.43	2.55
Romania	0.99	1.43	0.13	−0.40	0.22
Greece	0.53	1.06	0.45	0.04	0.01
Brazil	0.35	0.66	1.03	−0.75	0.62
Poland	0.87	0.87	0.53	0.50	−0.90
Germany	0.92	0.28	0.23	0.42	−0.38
Italy	0.81	0.06	0.30	−0.02	−0.08
United Kingdom	0.44	0.65	0.60	−0.17	−0.73
China	0.13	−1.05	0.21	0.91	0.55
France	0.81	−0.83	0.62	−0.31	−0.98
Netherlands	0.33	−0.33	0.05	0.29	−1.64
Sweden	0.08	−0.37	0.14	−0.41	−1.15
Indonesia	−0.60	−0.78	−1.30	−0.60	0.60
Japan	−0.98	−1.29	−0.09	0.66	−1.10
Nigeria	−1.30	−0.84	−1.63	−1.19	0.89
India	−1.39	−1.13	−1.66	−0.77	−0.67
Kenya	−2.16	−0.82	−1.54	−1.24	0.02
Ethiopia	−1.82	−1.53	−1.66	−1.31	−0.32

**Table 4:** Pairwise Pearson correlation coefficients among the five standardised pressure indicators, with bootstrap 95% confidence intervals (2000 resamples, percentile method) (n = 20 countries).

Indicator Pair	Pearson r	95% CI (Bootstrap, n = 2000)
Caloric supply—Obesity	0.74	[0.51, 0.90]
Caloric supply—Meat consumption	0.87	[0.71, 0.94]
Caloric supply—CO_2_ emissions	0.60	[0.22, 0.81]
Caloric supply—Food waste	0.05	[−0.30, 0.38]
Obesity—Meat consumption	0.73	[0.51, 0.89]
Obesity—CO_2_ emissions	0.46	[−0.04, 0.76]
Obesity—Food waste	0.43	[0.03, 0.67]
Meat consumption—CO_2_ emissions	0.74	[0.44, 0.90]
Meat consumption—Food waste	0.17	[−0.30, 0.49]
CO_2_ emissions—Food waste	0.17	[−0.47, 0.60]

**Table 5:** PCA loadings and indicator correlations with the first two principal components (n = 20 countries).

Indicator	PC1 Loading	PC2 Loading	Corr. with PC1	Corr. with PC2
Daily caloric supply	0.51	−0.25	0.90	−0.26
Obesity prevalence	0.48	0.24	0.86	0.25
Meat consumption	0.53	−0.16	0.95	−0.17
CO_2_ emissions/capita	0.44	−0.16	0.78	−0.16
Food waste/capita	0.18	0.91	0.33	0.93

Note: a bootstrap stability check (2000 resamples, percentile method) confirms these results are not an artefact of the specific 20-country sample. The PC1 and PC2 explained-variance ratios are stable at 63.6% (95% CI [50.4%, 73.7%]) and 21.9% (95% CI [16.0%, 30.5%]), respectively, and food waste’s loading on PC2 remains stable at 0.832 (95% CI [0.518, 0.965]), consistent with the base values reported above. No values in Table 5 change as a result of this check (Section Robustness Checks).

**Table 6:** Principal component scores (PC1, PC2) for each of the 20 countries (n = 20 countries).

Country	PC1 Score	PC2 Score	Tier
United States	3.75	0.34	Tier 1
Australia	2.82	0.84	Tier 1
Mexico	1.13	2.62	Tier 1
Romania	1.13	0.35	Tier 1
Greece	1.04	0.05	Tier 1
Brazil	0.83	0.59	Tier 1
Poland	1.20	−0.99	Tier 1
Germany	0.84	−0.61	Tier 2
Italy	0.57	−0.30	Tier 2
United Kingdom	0.65	−0.69	Tier 2
China	0.17	0.03	Tier 2
France	0.02	−1.35	Tier 2
Netherlands	−0.14	−1.71	Tier 2
Sweden	−0.46	−1.11	Tier 3a
Indonesia	−1.52	0.81	Tier 3b
Japan	−1.11	−1.19	Tier 3a
Nigeria	−2.29	1.39	Tier 3b
India	−2.60	−0.14	Tier 3b
Kenya	−2.85	0.81	Tier 3b
Ethiopia	−3.18	0.27	Tier 3b

**Table 7:** Differentiated governance and policy instruments mapped to the four typology cells identified in this study.

Typology Cell	Study Countries	Plausible Primary Driver *	Primary Instrument Family	Real-World Example
Tier 1—high-abundance, high-pressure	US, Australia, Mexico, Romania, Greece, Brazil, Poland	Retail over-provision + discretionary household waste amid abundance	Regulatory ban on destruction + metered household pricing	Loi Garot (France, 2016); volume-based food waste fee (South Korea) [52,53]
Tier 2—intermediate	Germany, Italy, UK, China, France, Netherlands	Mixed retail/household, moderate abundance	Fiscal incentives + consumer information/labelling	Gadda Law (Italy, 2016); EU date-labelling reform/Farm to Fork [54,55]
Tier 3a—affluent-moderate	Sweden, Japan	Already low; norm maintenance	Public education; benchmark/comparator case	National dietary and food-citizenship education campaigns [57,58]
Tier 3b—lower-income/constrained	Indonesia, Nigeria, India, Kenya, Ethiopia	Plausibly upstream, infrastructure-driven loss (hedged hypothesis, Section 5.1)	Post-harvest infrastructure and cold-chain investment	FAO/development-bank food-loss-reduction programmes [37,51]

* Note—Not causally tested.

## Data Availability

No new primary data were generated. All indicators analysed are publicly available: daily dietary energy supply and meat consumption (FAO, via OWID, https://ourworldindata.org/grapher/daily-per-capita-caloric-supply (accessed on 2 September 2026) and https://ourworldindata.org/grapher/daily-meat-consumption-per-person) (accessed on 2 September 2026); obesity prevalence (WHO Global Health Observatory, via OWID, https://ourworldindata.org/grapher/share-of-adults-defined-as-obese) (accessed on 2 September 2026); CO_2_ emissions per capita (Global Carbon Project, via OWID, https://ourworldindata.org/co2/country/) (accessed on 2 September 2026); arable land per capita (World Bank World Development Indicators, indicator AG.LND.ARBL.HA.PC); and food waste per capita (UNEP Food Waste Index 2024, via OWID, https://ourworldindata.org/grapher/food-waste-per-capita) (accessed on 2 September 2026). The compiled 20-country dataset, the SPI, the standardisation and correlation computations, and the PCA loadings and scores underlying Table 1 and Table 3, Table 4, Table 5 and Table 6 and Figure 2 and Figure 3 are reported in full in the manuscript and its Supplementary Materials.

## Supplementary Materials

The following supporting information can be downloaded at https://www.mdpi.com/article/10.3390/foods15213822/s1, Table S1: Reference year, data-source vintage, and confidence classification for each of the five pressure indicators (daily caloric supply, meat consumption, obesity prevalence, CO2 emissions per capita, and food waste per capita), by country (n = 20); for caloric supply, meat consumption, obesity, and CO2 emissions, each country’s reference year is additionally cross-checked against the current Our World in Data (OWID) vintage, with a match confirmed within a ±3% relative threshold, while food waste reference year and confidence classification are taken directly from the UNEP Food Waste Index Report 2024 country appendix, with a separate confidence note for the non-household (retail and food-service) portion of the combined per-capita figure; Supplementary_Data.xlsx: a spreadsheet reproducing the 20-country raw dataset and all derived computations reported in Table 1, Table 3, Table 4, Table 5 and Table 6 of the main text, built with live spreadsheet formulas so that every derived value can be traced back to the raw data cell by cell. All underlying data and computations are reported in full in the body of this manuscript as Table 1, Table 3, Table 4, Table 5 and Table 6 (raw dataset and SPI, z-score standardisation, pairwise correlation matrix with bootstrap confidence intervals, PCA loadings, and per-country PCA component scores, respectively), each captioned with the equation from Section 3 that generated it; Table 2 additionally reports the SPI robustness checks described in Section Robustness Checks. For those who wish to verify or re-run these computations directly, the following sup-porting information is additionally available: Supplementary_Data.xlsx, a spreadsheet reproducing the same 20-country raw dataset, the z-score standardisation underlying Equation (1), the pairwise correlation matrix (Equation (3)) and the PCA loadings and scores (Equations (4) and (5)) underlying Figure 2 and Figure 3, built with live spreadsheet formulas so that every derived value can be traced back to the raw data cell by cell.

## Abbreviations

The following abbreviations are used in this manuscript:

SPI	Systemic Pressure Index
PCA	Principal Component Analysis
OWID	Our World in Data
FAO	Food and Agriculture Organization of the United Nations
WHO	World Health Organization
UNEP	United Nations Environment Programme
GHG	greenhouse gas
CO_2_	carbon dioxide
EU	European Union
